# The Most Interesting Hospital in the World

**Published:** 1891-12-05

**Authors:** 


### The Most Interesting Hospital in the World.

By Our Special Commissioner.

(*Concluded from page* 101).

### Its Organisation.

The principle upon which the Johns Hopkins Hospital is organised is one which must commend itself to all sound administrators. The policy of the trustees has been to pay every officer adequately, and then, by careful investigation and inquiry, to select for each post the very best and most experienced person who was available to fill the office in question. Dr. Hurd, the Superintendent, in his first report, shows that the organisation differs in some essential features from that of other general hospitals in the United States. The service is divided into three distinct departments—medical, surgical, and gynæcological, each of which is placed under a responsible chief, with a continuous service. The heads of these departments are non-resident, but arrangements are made for them to give as much time to the work of the hospital as the necessities of the patients demand; that is to say, that every officer of the hospital receives adequate remuneration, and binds himself to make its work his first consideration during every hour in each day which it may be necessary to devote to the service.

Each department has a responsible resident physician, who has had a long and varied experience in a general hospital, and who is abundantly able to fill the place of the chief of the department when he is absent from the hospital. Each resident physician has a staff of assistants, who give aid in case taking, surgical operations, clinical notes, and examination of urine, sputum, blood, etc., and also in dispensary work. The assistant physicians, surgeons, and gynæcologists are resident in the hospital. The dispensary has a chief, who directs and arranges the work of the different departments, and each department in turn is under the special direction and control of a responsible head, who takes care of the work, and has a continuous service. Each head of the dispensary department has as many assistants as the proper work of the department requires, whose medical work he directs and controls.

The nursing work of the hospital is under the charge of Miss Hampton, Superintendent of Nurses, who also acts as the principal of the training school. She is responsible for the management of the nurses' home and instruction of nurses; she selects and accepts probationers, prescribes the course of study, and provides all nurses with work.

The purchase and delivery of provisions to the cook, and distribution of service or food, are placed in the hands of a purveyor, who is made responsible for this branch of the work. The care of the rooms in the building, and the overseeing of the work of the laundry is in charge of the Matron, whose duty it is to purchase bedding, dry goods, clothing, household and laundry supplies. In addition to these officers there is a controller of accounts to supervise the receipts of money and the payment of bills, and an apothecary, who purchases medicine, and prepares and delivers prescriptions; a supervisor of grounds, who looks after all outside matters about the buildings, and an engineer, who has the care and oversight of the engines, boilers, filters, pumping apparatus, machinery, warming and ventilating apparatus, water tanks, sewers, water closets, elevators, steam cooking apparatus, water, gas, electrical, and steam distribution.

The entire organisation is under the control, supervision, and management of the Superintendent, Dr. Henry M. Hurd, whose accuracy, administrative capacity, and warm interest and pride in the hospital combine to make him an ideal official. He is supported by the heads of the various departments and by the staff, who labour zealously and constantly with the one aim of securing to the hospital a high standing for scientific work, faithful medical service, good nursing, and efficient administration.

### Special Features.

We have not space to go into all the varied and many matters which combine to make the Johns Hopkins Hospital the most interesting hospital in the world. The medical, surgical, gynæcological, pathological, and post-graduate departments are each deserving of special notice. We shall hope to deal with some or all of them on a future occasion. If criticism were our object, we might express a preference for arched and tiled corridors, and the hope that it might be possible to make arrangements to secure a crisper administration of the kitchen department. Everything that care, intelligence, and thought can secure to make the food appetising and attractive to the patient is already done. Owing, no doubt, to the difficulty of finding servants of the highest type in the United States, the kitchen at present lacks incisive cleanliness in its appearance, utensils, and arrangements. We do not wish it to be understood that the kitchen arrangements are uncleanly, but merely that to the eye of the visitor something is wanting in this department to brighten and present an attractive appearance which shall commend its administration to the notice of the trained inspector.

The wards are of two forms, rectangular and octagonal. The system of ventilation introduced is probably the most perfect artificial system possessed by any institution. The whole of the arrangements of the rectangular wards are so organised that the staff cannot interfere with their action. In the octagonal wards, however, the nurses have it in their power to interfere with the ventilation, and at the time of our visit we found that, although both the rectangular and octagonal wards had the same temperature, nearly 69 degrees, the atmosphere of the former was fresh and invigorating, while that of the latter left something to be desired. A little inquiry revealed the cause. In the octagonal wards the nurses of their own accord had closed the ventilators, so that the atmosphere was unduly impregnated with products which would have been absent had the arrangements been identical with those of other parts of the building.

It is the experience of everyone who has studied the subject of ventilation, that where artificial means are employed, to secure success, care must be taken to so arrange the apparatus that no member of the staff can interfere with its action, and that the admission or exclusion of air, as well as the amount of heat to be employed, must be left to the trained experience of a special officer charged with the duty of continuously studying the conditions of the wards, and also of the outside atmosphere, not only every day, but from hour to hour. Unless this is done, the most perfect system will prove, in practice, a failure, because no subject is so little understood by the ordinary nurse, and, indeed, by the majority of people, as that of ventilation, not only by artificial, but by natural means too.

It is a matter of interest, on account of the large number of coloured people residing in Baltimore, that the experience of the hospital authorities there is that they cannot be admitted to the hospital unless the managers provide separate wards for the accommodation of this class of the population. Prejudice is a difficult thing to kill, and although American hospital authorities are able to mix paid and unpaid patients in a common ward without protest or difficulty, they find it impossible to treat coloured people and white people together without causing difficulties which cannot be coped with.

The Nursing Department.

The nursing home, the nursing staff, and the system of training adopted, are all admirable. They have been modelled upon the best types known to the initiated, and as Miss Hampton is one of the ablest and most intelligent of Superintendents, this department of the hospital is so efficient as to leave little or nothing to be desired. One feature connected with it is both novel and worthy of imitation. Pupil nurses are now taught the science and art of cooking for the sick. This department is under the charge of Miss Boland, a graduate of the Boston Cooking School, who was appointed to organise the cooking school for this hospital last year. The diet kitchen has been fitted up for the purpose in connection with the general kitchen of the hospital. Miss Hampton has written an excellent account of this work, from which we extract the following information :—

The organisation of the cooking school resembles that of a ward with its head nurses and pupils, the teacher corresponding to the head nurse. Two pupils are sent to her for a month at a time. Their hours of duty are from 7.30 a.m. to 5.30 p.m. The first hour and a-half of the day is spent in the two private wards, where each pupil takes charge of the preparations for breakfast, makes the toast, arranges the trays daintily, and gets everything in readiness for breakfast, which arrives from the general kitchen at eight o'clock. They then serve the trays and leave the wards at 9 a.m., going directly to the cooking school kitchen, which is a small room conveniently situated and easily accessible to all the wards. There they meet the teacher and the day's instruction begins. The course includes the general subject of the preparation of beef essences, beef teas, broiled meats, steaks, chops, and birds, gruels, porridges, mushes, drinks, jellies, toasts, soups, broths, oysters, eggs, potatoes, custards, sherbets, creams, frozen fruits, cordials, salads, koumiss, and all simple dishes, such as baked apples, plain boiled rice, and the like. The beaf tea, chicken broth, and mutton broth for use in the entire hospital are made each morning, and in addition practical demonstration of the process of making dishes selected from the above schedule are given by the teacher every forenoon. The method of preparing about one hundred and fifty different articles of sick diet is taught during the course, and each article is made at least three times by the pupils themselves. The greater part of the day's cooking is distributed among the various wards at noon.

The afternoon hours are chiefly devoted to theoretical teaching, which includes, for example, talks on the effect of heat on food, the effect of cold, fire, the chemistry of foods, oxygen, the composition of air, water, the cooking of food, the mineral and organic matter in the same, albumen, and methods of serving food. All notes, lectures, and recipes are written out in full. Towards the end of the month a practical test is given of the proficiency of each pupil by requiring her to make as large a number of dishes as possible, without aid from either teacher or notes. An oral examination is given at the end of the course, and this is followed at the end of another month by a written test.

There can be no doubt that this novel feature of the Johns Hopkins Hospital is worthy of imitation by every nurse-training school in the world. It is essentially necessary that every private nurse and every district nurse should have some knowledge of invalid cookery. A personal visit to this school has shown us that every detail is carefully thought out, and that any nurse who has had the advantage of going through a course of training here must necessarily be much more efficient and useful, and capable of discharging adequately all the duties of her calling than those of her sisters who have lacked similar opportunities.

We hope to give a full account of the working of this branch of the Johns Hopkins Hospital, which cannot fail to interest the authorities of nurse-training schools, as well as the nurses themselves.

Of course, we have touched upon a very few points. Many more remain to be dealt with, but to do justice to this great institution, to understand the amount of fruitful thought which has been put into its organisation, and to realise its value, any one ought to take three weeks' holiday across the Atlantic, and devote four or five days to a careful investigation on the spot.

